# Prokineticin 2 neurons form diverse subpopulations in the suprachiasmatic nucleus and rely on VPAC2-signaling for diurnal rhythmicity

**DOI:** 10.3389/fphys.2025.1619673

**Published:** 2025-07-15

**Authors:** Ida Stangerup, Birgitte Georg, Jens Hannibal

**Affiliations:** ^1^ Department of Clinical Biochemistry, Copenhagen University Hospital - Bispebjerg and Frederiksberg, Copenhagen, Denmark; ^2^ Department of Clinical Medicine, Faculty of Health and Medical Sciences, University of Copenhagen, Copenhagen, Denmark

**Keywords:** Immunohistochemistry, AVP, VIP, NMS, PAC1, EGR1, FOS

## Abstract

**Introduction:**

Prokineticin 2 (PK2) is believed to function as an output molecule, relaying circadian rhythms of behavior and physiology from the suprachiasmatic nucleus (SCN). The expression of PK2 in the SCN is primarily driven by the molecular clock, oscillating with high levels early-mid day and low levels during night. Furthermore, light at night induces the expression of PK2. Until recently, the absence of a reliable PK2 antibody has hindered characterization of PK2 neurons in the SCN, including whether they constitute a phenotypically homogeneous population or comprise distinct subtypes, some potentially light-responsive. Hence, the aim of this study was to characterize PK2 neurons in relations to light-responsiveness and neuropeptidergic markers of the core-shell division.

**Methods:**

Double and triple immunohistochemistry of PK2 together with phenotypical neuropeptide/receptor markers of the core-shell division was performed at zeitgeber time (ZT) 8. Light-responsiveness of PK2 SCN neurons was evaluated using FOS and EGR1 ZT18, 2 h following a 30-min light-pulse stimulation. Data were visualized and processed using confocal microscopy. Moreover, PK2 mRNA was evaluated over a 12:12 light-dark cycle in both wildtype and VIP type 2 receptor (VPAC2) knockouts mice, using qPCR.

**Results:**

The majority of PK2 neurons were located in the shell, constituting a subpopulation of vasopressin (AVP) and neuromedin S (NMS) neurons. Few PK2 neurons were found in the ventral core, constituting a subpopulation of vasoactive intestinal polypeptide (VIP) and NMS neurons. PK2 shell neurons expressed the VPAC2 receptor, and in its absence, diurnal rhythmicity of PK2 mRNA was abolished. In addition, the PAC1 receptor—specific for pituitary adenylate cyclase-activating polypeptide (PACAP), one of two neurotransmitters of the retinohypothalamic tract—was found on some PK2 neurons. Moreover, nighttime light-pulse stimulation broadly induced FOS and EGR1 immunoreactivity throughout the SCN but only in few PK2 neurons.

**Conclusion:**

PK2 SCN neurons are heterogeneous yet highly dependent on intact VIP-VPAC2 signaling to maintain a coherent diurnal expression pattern. A neuroanatomical and functional basis suggest two potential pathways for regulating PK2 expression: a diurnal mechanism involving VIP signaling through the VPAC2 receptor and a direct light-mediated pathway via retinal innervation through the PAC1 receptor.

## Introduction

The endogenous circadian rhythm of the suprachiasmatic nucleus (SCN) is approximately but not exactly 24 h and thus requires synchronization with the astronomical day—a process known as entrainment (Golombek and Rosenstein, 2010). The most important external cue (zeitgeber) for entrainment is light, which is detected by melanopsin-expressing intrinsically photosensitive retinal ganglion cells (ipRGCs) in the retina, transmitting signals via the retinohypothalamic tract (RHT) to the SCN (Golombek and Rosenstein, 2010; Hannibal, 2021). The ipRGCs utilizes two neurotransmitters, glutamate and pituitary adenylate cyclase-activating polypeptide (PACAP) for the transmission of light signals (Hannibal, 2021). The approximately 20,000 SCN neurons form a cohesive network, which anatomically and functionally can be divided into two distinct regions: the core and the shell (Moore et al., 2002). The core constitutes the retinorecipient part of the SCN with neurons expressing the PACAP type 1 receptor (PAC1) as well as neurons expressing vasoactive intestinal polypeptide (VIP), a directly light responsive neuron population (Riedel et al., 2023; Hannibal et al., 2017). The majority of neurons in the shell express rhytmically regulated neuropeptides, such as vasopressin (AVP) and prokineticin 2 (PK2), which appear to act as principal transmitters of circadian timekeeping signals from the SCN to other brain regions, tissue and organs throughout the body (Mieda et al., 2015; Evans et al., 2015). PK2, in particular, has been proposed as a key output molecule of the SCN, involved in transmitting circadian rhythms of behavior—most notably locomotor activity and sleep (Cheng et al., 2002; Prosser et al., 2007; Zhou and Cheng, 2005; Li et al., 2006; Hu et al., 2007). Furthermore, recent evidence suggests that PK2-signaling may also influence the coordinated timing of neuronal populations within the SCN, as selective activation of cells expressing PK2 and its’ receptor within an otherwise circadian-incompetent network can trigger oscillations and establish the network period (Morris et al., 2021).

Both PK2 mRNA and protein exhibit diurnal rhythmicity, with peak expression early-mid day, where nocturnal animals are inactive (Cheng et al., 2002; Lambert et al., 2005; Masumoto et al., 2006; Stangerup et al., 2025). PK2 mRNA is nearly undetectable during large parts of the night, whereas PK2 protein remains present throughout the night, although at lower levels (Cheng et al., 2002; Stangerup et al., 2025). The expression of PK2 is primarily regulated by the molecular clock, but it can also be modulated by light, as nocturnal light exposure rapidly induces PK2 mRNA expression in the SCN (Cheng et al., 2002; Cheng et al., 2005). In mice, light exposure during the early night induces phase delays, while late-night exposure results in phase advances—both linked to changes in PK2 mRNA expression, potentially supporting a role for PK2 in photoentrainment (Cheng et al., 2005). Neuroglobin (Ngb), a neuron-specific, oxygen-binding globin, is expressed in light-responsive neurons of the SCN core and has been implicated in the modulation of light-induced phase delays (Riedel et al., 2023; Hundahl et al., 2012). However, potential co-expression of PK2 and Ngb within the same SCN neurons has not yet been investigated.

VIP-signaling from the ventral core to shell neurons occur via the VIP type 2 receptor (VPAC2). VIP and the VPAC2 receptor are crucial for core clock function, as mice lacking either VIP or the VPAC2 receptor exhibit disrupted SCN rhythmicity (Harmar et al., 2002; Piggins and Cutler, 2003; Colwell et al., 2003; Hannibal et al., 2011). Specifically, VPAC2-deficient mice display a profoundly disrupted circadian phenotype, where locomotor activity is primarily governed by masking, and core body temperature rhythm is phase-advanced by approximately 6 h and uncoupled from the activity and heart rate profile (Hannibal et al., 2011). Additionally, in the absence of VIP alone, PK2 appears to lose its characteristic diurnal rhythmicity, suggesting an interaction between the two neuropeptide axes, although the underlying mechanisms remain poorly understood (Bedont et al., 2018).

Using immunohistochemistry (IHC), we have recently shown that more than 80% of PK2 shell neurons are clock cells, as evaluated by PER2 expression (Stangerup et al., 2025). Furthermore, RNA sequencing studies suggest that the majority of PK2 neurons represent a subpopulation of AVP neurons (Morris et al., 2021; Wen et al., 2020; Chen et al., 2017). However, the extent to which PK2 is co-expressed with neuropeptidergic neurons and their receptors in both the core and shell regions remains unclear. RNA sequencing studies provide valuable information from single neurons but fail to capture the details of SCN topology.

The aim of this study was to characterize distinct phenotypes of PK2 neurons in relation to core and shell division markers using IHC. Given that we found pronounced co-expression of PK2 and VPAC2 in neurons of the shell, we next investigated PK2 mRNA expression in mice lacking the VPAC2 receptor—a model with a dysfunctional clock. Additionally, we assessed the light responsiveness of PK2 neurons by examining FBJ osteosarcoma oncogene (FOS) and early growth response 1 (EGR1) protein expression following nighttime light stimulation.

## Materials and methods

### Ethics

The study was conducted in compliance with the Laboratory Animal Care guidelines as mandated by the Danish Law on Animal Experiments (LBK NR1597, 8 July 2021) and was approved by Dyreforsøgstilsynet under the Ministry of Food, Agriculture, and Fisheries, Denmark (license number 2021-15-0201-00929, issued to Jens Hannibal).

### Animals

Animals were housed in individual cages under standardized laboratory conditions with a controlled 12-h light:dark cycle (12:12 LD) and had *ad libitum* access to water and food (Altromin 1324, Altromin Specialfutter, Germany). All animals were at least 10 weeks of age prior to inclusion in experimental protocols. To assess PK2 co-expression with markers of the core-shell divisions, IHC was performed zeitgeber time (ZT) 8 (ZT0: lights ON; ZT12: lights OFF) on PACAP wildtype (PACAP+/+) mice bred on a 129/Sv background. To assess light-responsiveness in PK2 neurons, IHC was performed on brains dissected at ZT18. A total of 30 animals were included for IHC experiments with each experiment conducted at least twice and performed on a minimum of three animals. Quantitative polymerase chain reaction (qPCR) was performed on 52 VPAC2 wildtype (VPAC2+/+) and 58 knockout (VPAC2 −/−) mice bred on a C57BL/6 background ZT4, ZT8, ZT12, ZT16, ZT20 and ZT24 with 7–11 animals included at each timepoint. Details on breading of the VPAC2−/− strain is described elsewhere (Fahrenkrug et al., 2018). Since no immediate evidence of obvious sex differences was observed, both male and female mice were included equally in all experiments.

### Light experiments

Mice received a 30-min white light pulse ZT16 delivered by fluorescence tubes placed on top of their home cages. Light intensity was measured using an Advantest Optical Power meter TQ8210 (MetricTest, Hayward, CA), and measurements were determined at settings of ∼514 nm giving 115 μW/cm^2^*s (∼300 lux). The animals were sacrificed at ZT18, 120 min after the light pulse initiation. Until sacrifice, animals were maintained in darkness.

### Tissue preparation

Mice for IHC were anesthetized prior to sacrifice via a subcutaneous injection of hypnorm and midazolam (0.1 mL per 10 g body weight). Transcardial perfusion was performed with heparinized phosphate-buffered saline (PBS; 15,000 IE/L, pH 7.2) for 3 min, followed by Stefanini fixative (2% paraformaldehyde, 15% picric acid in 0.1 M PBS, pH 7.2) for 15 min. Brains were then dissected, postfixed overnight in Stefanini fixative, and cryoprotected in 30% sucrose before freezing at −80°C. Mice for qPCR were decapitated whereafter brains were rapidly removed and immediately frozen at −80°C.

Brains were cut in coronal sections on a Leica CM 3050 cryostat at −15°C in thickness of 40 µm for free floating IHC. For qPCR, brains were cut on the cryostat in a thick 300 µm coronal section followed by dissection of the SCN with a scalpel (Fahrenkrug et al., 2005).

### Immunohistochemistry (IHC)

IHC was conducted on 40 µm thick free-floating brain sections as previously described (Hannibal et al., 2017; Hannibal et al., 2022). Pretreatment in antigen retrieval (1.5 h at 80°C in citrate buffer (pH 6, Dako EnVision FLEX, Cat# GV805) followed by blocking of endogenous peroxidase activity (1% H2O2 in 1× PBS) and blocking with 5% normal donkey serum (Jackson Immunoresearch Laboratories, Cat# 017-000-121) preceded incubation with primary antibodies. For EGR1 staining, sections were post-fixed in 4% paraformaldehyde at room temperature for 120 min before undergoing antigen retrieval.

Tissue sections were incubated with primary antibodies at 4°C for 2 days, followed by overnight incubation with secondary antibodies. PK2 detection was carried out using the ENVISION^©^ amplification system and visualized with Alexa-488 conjugated tyramide. The PK2 antibody is characterized in previous work (Stangerup et al., 2025). Detection of VPAC2, PAC1, cholecystokinin (CCK), neuroglobin (Ngb) and EGR1 were visualized by biotinylated antibody subsequently followed by incubation with Avidin-Biotin-peroxidase Complex (ABC) (Vector Laboratories, Cat# PK-6100), biotinylated tyramide and streptavidin conjugated Alexa594. The VPAC2, PAC1, CCK, Ngb and EGR1 antibodies have been characterized previously (Hannibal et al., 2017; Hundahl et al., 2010; Hannibal et al., 2010; Riedel et al., 2020). The CCK antibody was kindly donated by Professor Jens F. Rehfeld, and the Ngb antibody kindly donated by Christian A. Hundahl. The FOS antibody was kindly donated by Dr. Phillip J. Larsen and has been characterized elsewhere (Woldbye et al., 1996). AVP, VIP, Neuromedin S (NMS) and FOS were visualized using a secondary antibody labeled with Alexa Fluor 647.

IHC involving primary antibodies raised in the same species were performed as previously described (Hannibal et al., 2022). A thorough titration of the primary antibody was performed prior to double-IHC using primary antibodies raised in the same species to ensure no crosstalk between the secondary antibodies used for visualization of the antigen. Further details on primary and secondary antibodies including information regarding dilutions, heat induced epitope retrieval, conjugates and suppliers are listed in Table 1.

**TABLE 1:** Primary antibodies, secondary antibodies and conjugates used for immunohistochemistry.

Primary antibodies	Clonality	Host	Dilution	HIER	Vendor/gift from	Cat#	RRID
PK2	P	Rabbit	1:500	GV805	Abcam	76747	AB_1524238
AVP	P	Guinea-pig	1:1000	GV805	Synaptic Systems	403 004	AB_2725758
CCK	P	Rabbit	1:120,000	GV805	J.F. Rehfeld	8007	AB_2762851
Ngb	P	Guinea-pig	1:2000	GV805	C.A. Hundahl	G	—
NMS	P	Rabbit	1:1000	GV805	US Biological	364376	—
VIP	P	Guinea-pig	1:500	GV805	EuroDiagnostica	BG-340	—
VPAC2	P	Rabbit	1:20,000	GV805	In-house raised	623-S	AB_2814676
PAC1	P	Rabbit	1:20,000	GV805	In-house raised	35J8	AB_2814675
EGR1	P	Rabbit	1:10,000	GV805	Santa Cruz	sc-189	AB_2231020
FOS	P	Rabbit	1:1000	GV805	P.J. Larsen	9412	—

Secondary antibodies	Used for	Host	Dilution	Target	Vendor	Cat#	RRID
Biotin-SP	CCK, VPAC2, PAC1, EGR	Donkey	1:800	Anti-rabbit	Jackson	711-066-152	AB_2340594
Biotin-SP	Ngb	Donkey	1:800	Anti-guinea-pig	Jackson	706-065-148	AB_2340451
EnVision©	PK2	Goat	1:2	Anti-rabbit	Agilent (Dako)	K4003	AB_2630375
Alexa Fluor IgG 647	AVP, VIP	Donkey	1:200	Anti-guniea-pig	Jackson	706-605-148	AB_2340476
Alexa Fluor IgG 647	FOS, NMS	Donkey	1:200	Anti-rabbit	Jackson	711-605-152	AB_2492288

Conjugates	Used for		Dilution		Vendor	Cat#	
TSA Biotin	CCK, Ngb, VPAC2, PAC1, EGR		1:100		AKOYA	SAT70001EA	
Alexa Fluor 594	CCK, Ngb, VPAC2, PAC1, EGR		1:500		Jackson	016-580-084	
TSA Alexa Fluor 488	PK2		1:250		Thermo Fisher	B40953	

PK2, prokineticin 2; AVP, vasopressin; CCK, cholecystokinin; Ngb, neuroglobin; NMS, neuromedin S; VIP, vasoactive intestinal polypeptide; vasoactive intestinal polypeptide type 2 receptor; PAC1, pituitary adenylate cyclase-activating peptide type 1 receptor; IHC, immunohistochemistry; HIER, heat-induced epitope retrieval; P, polyclonal; GV805, Citrate buffer pH6. Vendors: Abcam, Agilent (original manufacturer Dako), AKOYA Bioscience, Jackson ImmunoResearch Labs, Santa Cruz Biotechnology, Synaptic Systems, Thermo Fisher Scientific.

### RNA extraction, cDNA synthesis and qPCR

Total RNA was extracted from each SCN using the Invisorb® Spin Tissue RNA Minikit (Invitek Diagnostics GmbH, Berlin, Germany), following the manufacturer’s protocol. cDNA was synthesized from approximately 250 ng RNA using the High-Capacity cDNA Reverse Transcription Kit (ThermoFisher Scientific, Waltham, Massachusetts, United States) in a final concentration of 5 ng/μL total reaction volume. A RNA pool from mouse hypothalamus was used to generate a large batch of cDNA, which was used to create the standard curves for both PK2 and β2-Microglobulin (B2m), the latter serving as the endogenous control, as described previously (Georg et al., 2007). The RNA input for the synthesis of the standard curve was five times that of the samples; consequently, 25 ng of RNA was used per µL of the total reaction volume. qPCR for PK2 was performed using TaqMan gene expression assay Mm07298039_m1 (ThermoFisher Scienticfic) spanning exon 1 and 2. Reactions were conducted in 20 µL containing cDNA corresponding to 10 ng of total RNA and TaqMan Universal PCR Master Mix with AmpErase® UNG (Thermo Fisher Scientific). Quantification of B2m was conducted by primers, probe and conditions described previously (Georg et al., 2007). Reactions were run on a StepOnePlus Real-Time PCR System (Thermo Fisher Scientific). PK2 mRNA levels were quantified using the StepOne Software v2.3 (Thermo Fisher Scientific) and expressed in arbitrary units. The amount of PK2 mRNA in each sample was normalized to B2m levels, quantified on the same plate. All reactions were performed in duplicates.

### Imaging and data processing

Fluorescence images were obtained using an iMIC confocal microscope (TILL Photonics, FEI, Germany) equipped with the following objectives: ×10, numerical aperture (NA) = 0.35; ×20, NA = 0.75; x40, NA = 1.3 and appropiate filter settings for detection of DAPI 405, Alexa 488, Alexa 594 and Alexa 647. Z-stacks captured using ×40 objectives were deconvoluted with AutoQuant X (version 3.04) prior to analysis in IMARIS® (version 10.2.0). Co-localization was determined using the co-localization module in IMARIS® and defined as the presence of two antigen-specific signals within the same pixel. Moreover, instances where VPAC2 or PAC1 receptor immunoreactivity co-localized with PK2 immunoreactivity close to the cell membrane were interpreted as likely being synaptic appositions. Brightness and contrast adjustments for all images were performed using Fiji (v. 1.48v) and/or IMARIS® and final image assembly was completed in Adobe Illustrator (version 16.0).

PK2 co-expression with AVP, VIP, and NMS were quantified by manual cell counting using the Cell Counter plugin module in Fiji as previously described (Riedel et al., 2023). Z-stacks were obtained from two animals and cell counting was performed on sections representing the rostral, mid, and caudal part of the SCN for quantitative analysis of PK2 co-expression with AVP and NMS. Given the sparse presence of VIP neurons in the caudal part of the SCN, quantification of PK2 and co-expression with VIP was only assessed on Z-stacks representing the rostral and mid part. Co-expression is reported as a percentage calculated based on the total number of cells counted across both animals. To evaluate diurnal rhythmicity in PK2 mRNA expression, qPCR data was fitted to a combined cosine and sine function as described by Nelson et al. using SAS statistical software package (SAS Grid Studio Enterprise Guide 7.1) (Nelson et al., 1979). The model fit determines, among other parameters, the ZT timepoint for PK2 peak expression, representing the apex of the fitted curve. To compare PK2 mRNA expression between the VPAC2 +/+ and VPAC2 −/− genotypes at specific timepoints, a two-way ANOVA was performed, followed by multiple unpaired t-tests with Bonferroni correction in GraphPad Prism (version 9.4.1 for Windows). For all statistics, a p-value <0.05 was considered significant. All graphs were made using GraphPad Prism.

## Results

### PK2 is co-expressed with AVP in neurons of the shell

**FIGURE 1:**
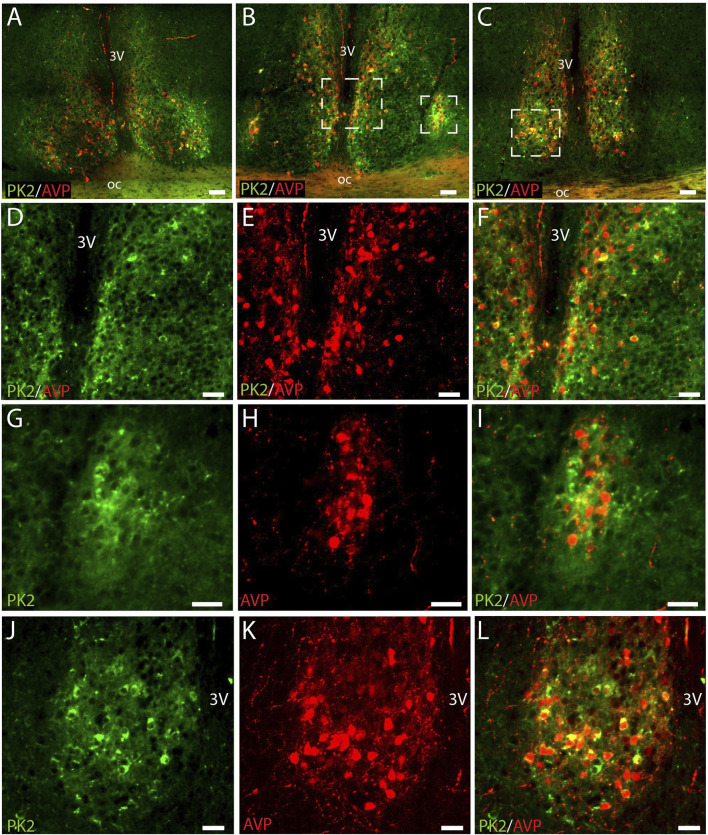
PK2 and AVP immunoreactivity at midday (ZT8) in the mouse SCN. **(A–C)** Show extensive PK2 (green) and AVP (red) immunoreactivity in the shell compartment of the rostral **(A)**, mid **(B)** and caudal **(C)** part of the SCN. **(D–F)** Represent the mid white frame in **(B)**, **(G–I)** the lateral white frame in **(B)** and **(J–L)** the central white frame in **(C)**. Note, how the majority of PK2 shell neurons in the mid and caudal part of the SCN co-express AVP indicated by the yellow color. PK2, prokineticin 2; AVP, vasopressin; SCN, suprachiasmatic nucleus; ZT, zeitgeber time; 3V, third ventricle; OC, optic chiasm. Scale bars: A–C, 50 μm; D–F, 30 μm; G–I, 25 μm; J–L, 50 μm.

**FIGURE 2:**
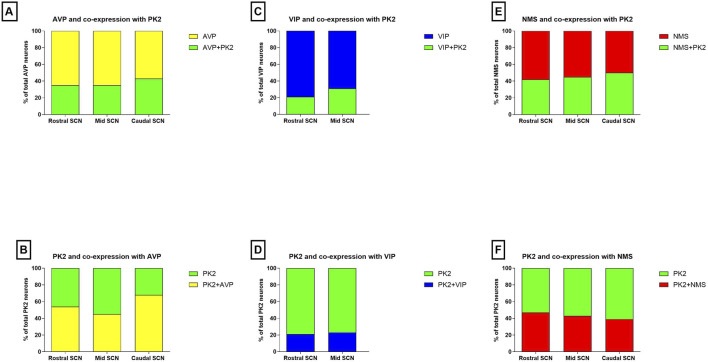
Co-expression of PK2 with AVP, VIP and NMS in the mouse SCN. **(A)** Shows the proportion of AVP neurons (yellow) co-expressing PK2 (green) and **(B)** shows the proportion of PK2 neurons (green) co-expressing AVP (yellow) in the rostral, mid and caudal SCN. **(C)** Shows the proportion of VIP neurons (blue) co-expressing PK2 (green) and **(D)** shows the proportion of PK2 neurons (green) co-expressing VIP (blue) in the rostral and mid SCN. **(E)** Shows the proportion of NMS neurons (red) co-expressing PK2 (green) and **(F)** shows the proportion of PK2 neurons (green) co-expressing NMS (red) in the rostral, mid and caudal SCN. Co-expression is assessed from double immunohistochemical stainings performed ZT8 and quantified by manual cell counting using the using the Fiji Cell Counter plugin module. Data represent the relative proportion of SCN neurons expressing the indicated neuropeptides, based on counts from two representative Z-stacks per section. PK2, prokineticin 2; AVP, vasopressin; VIP, vasoactive intestinal polypeptide; NMS, neuromedin S; SCN, suprachiasmatic nucleus; ZT, zeitgeber time.

We have recently shown that PK2 neurons are primarily located in the shell throughout the rostro-caudal extension of the SCN (Stangerup et al., 2025). AVP is the predominant neuropeptide in the SCN shell, and double-IHC experiments revealed, that PK2 neurons constitute a large subpopulation of AVP-expressing shell neurons throughout the entire SCN (Figure 1). Of the total number of AVP neurons, 35% in the rostral (86 out of 245), 35% in the mid (107 out of 302) and 43% in the caudal (169 out of 393) SCN co-expressed PK2 (Figure 2A; Supplementary Table S1). Conversely, of the total number of PK2 neurons, 54% (86 out of 160) in the rostral SCN, 45% (107 out of 236) in the mid SCN, and 68% (169 out of 249) in the caudal SCN co-expressed AVP (Figure 2B; Supplementary Table S1).

### The VPAC2 receptor is co-expressed in PK2/AVP neurons of the shell

**FIGURE 3:**
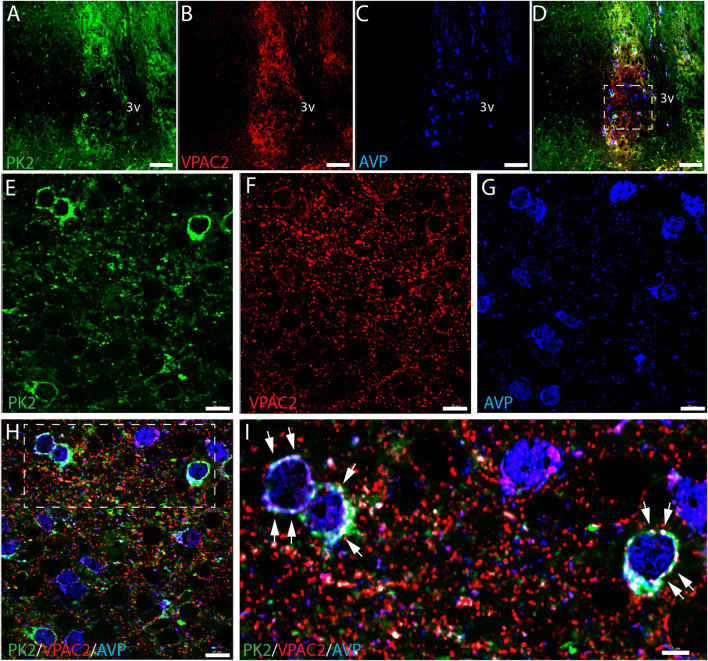
PK2, VPAC2, and AVP immunoreactivity at midday (ZT8) in the mouse SCN. **(A–I)** Show the results of triple immunostaining of PK2 (green), VPAC2 (red) and AVP (blue) in the mid SCN and how all three markers are broadly expressed in the mouse SCN. **(E–H)** Represent the white frame in **(D,I)** represents the white frame in **(H)**. In image **(D,H,I)** all three markers are presented together whereas image **(A,E)** solely show PK2, **(B,F)** VPAC2 and **(C,G)** AVP. In image **(H,I)** co-localization between PK2 and VPAC2 has been calculated and are illustrated by the white dots. Note, in the higher magnification **(I)**, how VPAC2 is located in the membrane of PK2 neurons as indicated by the white arrows and that these PK2 neurons also express AVP. PK2, prokineticin 2; VPAC2, vasoactive intestinal polypeptide receptor 2; AVP, vasopressin; SCN, suprachiasmatic nucleus; ZT, zeitgeber time; 3V, third ventricle; OC, optic chiasm. Scale bars: A–D, 50 μm; E–H, 10 μm; I, 5 μm.

VPAC2 is widely expressed in the shell, being target for VIP fibers originating from the ventral core (Hannibal et al., 2019). Triple-IHC revealed that PK2 neurons in the shell co-express the VPAC2 receptor throughout the rostro-caudal extension of the SCN and, moreover, represent a subpopulation of AVP neurons (Figures 3A–G). VPAC2 immunoreactivity appeared as dot-like staining, most likely representing synaptic complexes on the somato-dendritic membranes of the positively stained neurons (Figures 3H,I). This subcellular localization of the VPAC2 receptor—being distributed across both the somatic membrane and neuronal processes—poses significant technical challenges for accurate quantification. As a result, it was not possible to reliably determine the proportion of PK2 and AVP neurons co-expressing the VPAC2 receptor.

### PK2 is not co-expressed with CCK in neurons of the shell

**FIGURE 4:**
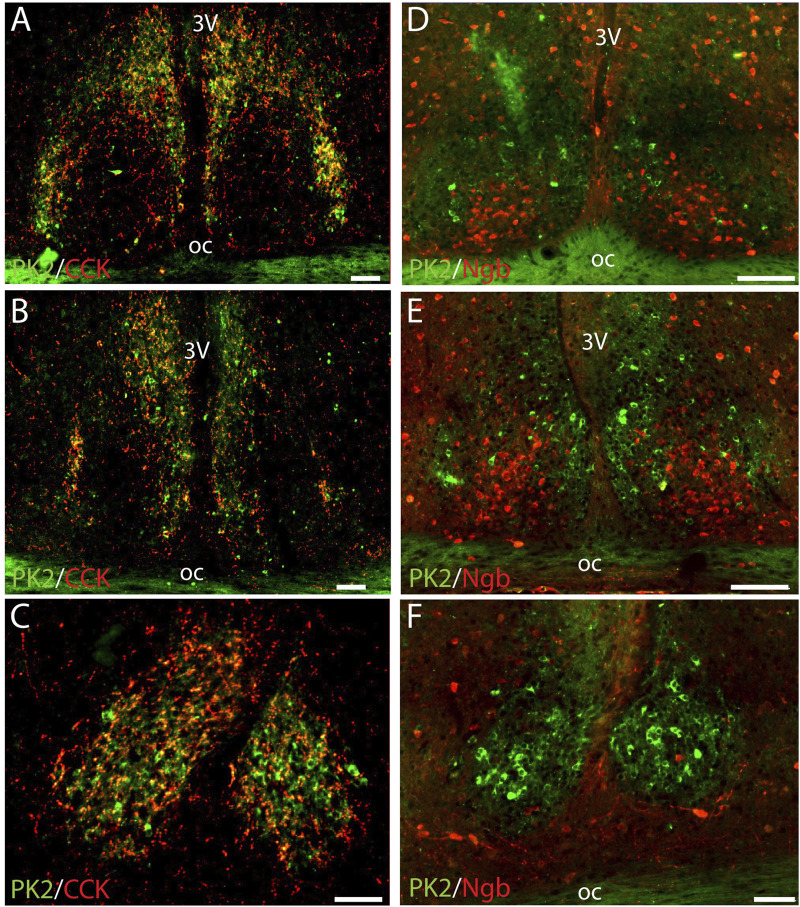
Double immunostaining for PK2 together with either CCK or Ngb at midday (ZT8) in the mouse SCN. **(A–C)** Show the results of double immunostaining of PK2 (green) and CCK (red) in the rostral **(A)**, mid **(B)** and caudal **(C)** part of the SCN, indicating no apparent co-expression of the two neuropeptides in nerve cell bodies. However, note that CCK nerve fibers closely surround PK2 neurons throughout the entire SCN shell with the yellow color indicating overlap between PK2 and CCK immunoreactivity - potentially representing synaptic contact between CCK and PK2 neurons. **(D–E)** Show the results of double immunostaining of PK2 (green) and Ngb (red) in the rostral **(D)**, mid **(F)** and caudal **(F)** part of the SCN. The image clearly illustrates how PK2 and Ngb neurons are two distinct SCN neuron populations with PK2 neurons primarily located in the shell and Ngb neurons extensively located in the central core. PK2, prokineticin 2; CCK, cholecystekinin; Ngb, neuroglobin; SCN, suprachiasmatic nucleus; ZT, zeitgeber time; 3V, third ventricle; OC, optic chiasm. Scale bars: A–E, 100 μm.

CCK is a neuropeptide expressed in a distinct population of SCN shell neurons with some being located lateral in the rostral part of the SCN shell (Figure 4A) (Hannibal et al., 2010). Despite a confined cluster of PK2-AVP neurons in the same part of the SCN (Figures 1B,I), no co-expression was found between PK2/AVP and CCK neurons in neither this part nor any part of the SCN (Figures 4A–C). However, CCK nerve fibers were readily visible in the SCN, forming a dense network surrounding PK shell neurons throughout the entire SCN (Figures 4A–C).

### PK2 is not co-expressed with Ngb in neurons of central core

Although PK2 neurons were primarily located in the shell, few were present in the core as well. We therefore assessed PK2 co-expression with Ngb, a phenotypical marker of the central core (Riedel et al., 2023). Double-IHC clearly demonstrated, that none of the PK2 neurons in the SCN core co-expressed Ngb (Figures 4D–F).

### PK2 is co-expressed with VIP in neurons of the ventral core

**FIGURE 5:**
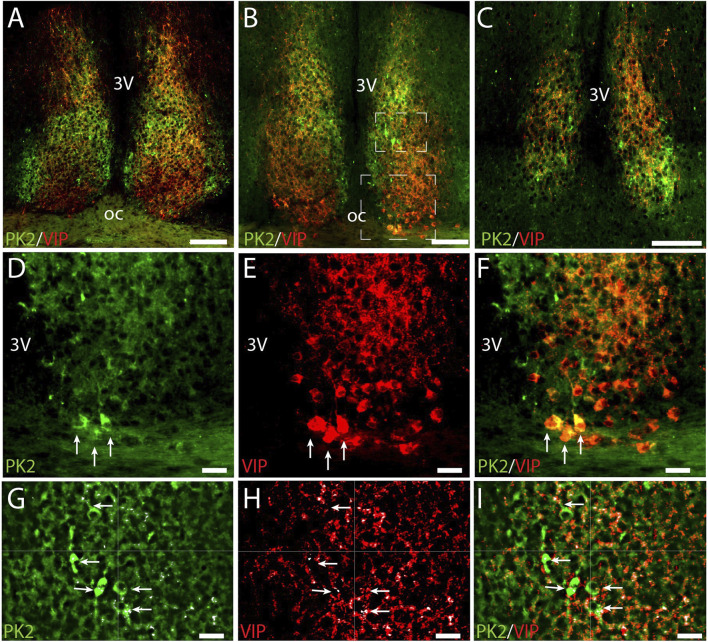
PK2 and VIP immunoreactivity at midday (ZT8) in the mouse SCN. **(A–C)** Show the results of double immunostaining of PK2 (green) and VIP (red) in the rostral **(A)**, mid **(B)** and caudal **(C)** part of the SCN. **(D–F)** Represent the big white frame in **(B)**. VIP neurons are extensively found in the ventral core **(E)**. Although the majority of PK2 neurons are located in the shell, a few are also present in the most ventral part of the SCN, where they co-express VIP, as highlighted by the white arrows **(D–F)**. **(G–I)** Represent the small white square in **(B)** showing VIP fibers closely surrounding PK2 neurons in the shell. In **(G–I)** the small white dots, highlighted by the white arrows, mark the pixels in where PK2 and VIP immunoreactivity are co-localizing. This co-localization was calculated using the IMARIS co-localization module and do most likely represent synaptic contacts between VIP and PK2 neurons. PK2, prokineticin 2; VIP, vasoactive intestinal polypeptide; SCN, suprachiasmatic nucleus; ZT, zeitgeber time; 3V, third ventricle; OC, optic chiasm. Scale bars: A–C, 100 μm; D–I, 20 μm.

To further characterize the minor population of PK2 neurons in the SCN core, we performed double-IHC together with VIP, a phenotypic marker of the ventral core, revealing co-expression in a subset of neurons (Figures 5A–F) (Riedel et al., 2023). Of the total number of VIP neurons, 21% (36 out of 173) in the rostral SCN and 31% (49 out of 158) in the mid SCN co-expressed PK2 (Figure 2C; Supplementary Table S2). Conversely, of the total number of PK2 neurons, 21% (36 out of 174) in the rostral SCN and 23% (49 out of 213) in the mid SCN co-expressed VIP (Figure 2D; Supplementary Table S2). As VIP neurons are sparsely present in the caudal SCN, no co-expression between PK2 and VIP was observed in this region. However, VIP fibers from the core were projecting dorsally to the shell, closely surrounding PK2 neurons (Figures 5A–C,G–I). Moreover, high-resolution photomicrograph analysis revealed apparent synaptic appositions between VIP-immunoreactive nerve fibers and PK2 neurons within the shell (Figures 5G–I).

### PK2 is co-expressed with NMS in both neurons of the core and shell

**FIGURE 6:**
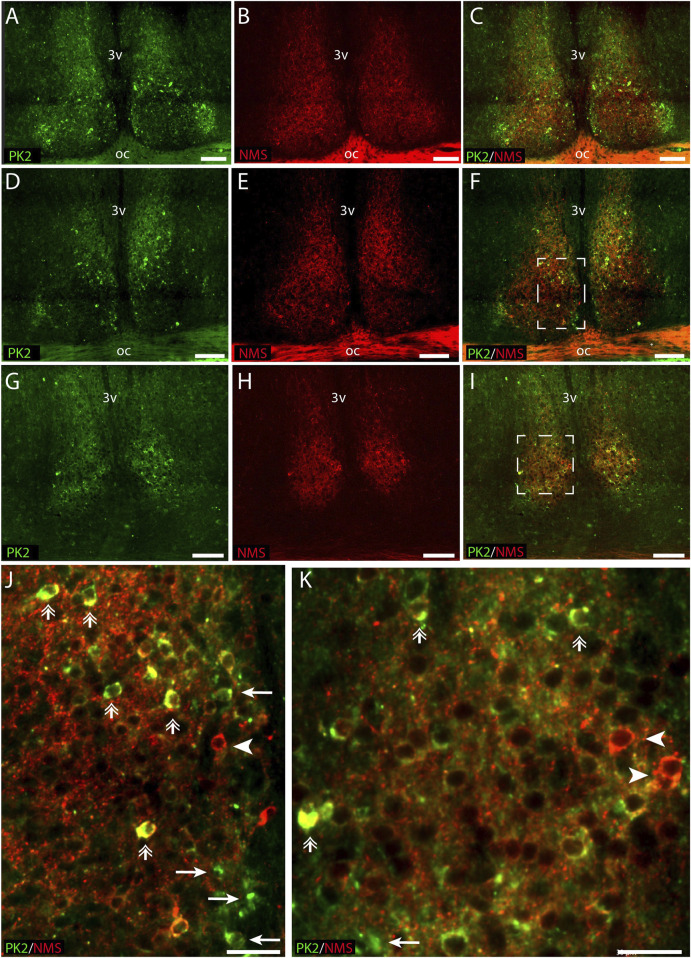
PK2 and NMS immunoreactivity at midday (ZT8) in the mouse SCN. **(A–I)** Show the results of double immunostaining of PK2 (green) and NMS (red) in the rostral **(A–C)**, mid **(D–F)** and caudal **(G–I)** part of the SCN. **(J)** Represents the white frame in **(F,K)** represents the white frame in **(I)**. Image **(A–I)** demonstrate how both PK2 and NMS are widely expressed in SCN neurons. PK2 is primarily localized in the shell but also present in the core, while NMS is distributed in both regions and forms a dense nerve fiber network, as indicated by the red dotting and particularly visible in the magnified images **(J,K)**. Moreover, **(J,K)** also demonstrate how a substantial group of neurons co-express PK2 and NMS as illustrated by the yellow color and highlighted by double arrows. Note, in image **(J,K)** that despite marked co-expression, neurons solely expressing either PK2 or NMS are seen as indicated by arrows and arrow heads, respectively. PK2, prokineticin 2; NMS, neuromedin S; SCN, suprachiasmatic nucleus; ZT, zeitgeber time; 3V, third ventricle; OC, optic chiasm. Scale bars: A–I, 50 μm; J–L, 30 μm.

RNA sequencing cluster analyses have previously grouped PK2 neurons with neurons expressing NMS, a neuropeptide found in both neurons of the core and shell (Morris et al., 2021; Wen et al., 2020). Double-IHC showed that a population of PK2 neurons located in both the core and shell co-express NMS, with NMS neurons appearing more densely distributed than PK2 in the core, whereas PK2 neurons seemed more prominent than NMS neurons in the shell (Figures 6A–I). Of the total number of NMS neurons, 42% (96 out of 228) in the rostral SCN, 45% (119 out of 262) in the mid SCN and 50% (71 out of 143) in the caudal SCN co-expressed PK2 (Figure 2E; Supplementary Table S3). Moreover, NMS nerve fibers were forming a dense network throughout the rostro-caudal extension of the SCN (Figures 6B,E,H). Of the total number of PK2 neurons, 47% (96 out of 203) in the rostral SCN, 43% (119 out of 274) in the mid SCN and 39% (71 out of 182) in the caudal SCN co-expressed NMS (Figure 2F; Supplementary Table S3).

### Loss of the VPAC2 receptor abolishes diurnal rhythmicity of PK2 expression

**FIGURE 7:**
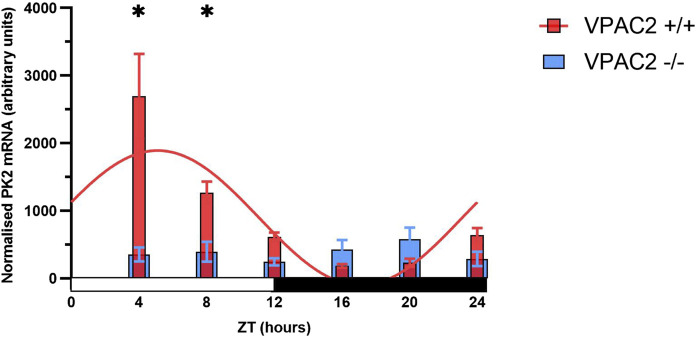
PK2 mRNA in arbitrary units in the SCN of the VPAC2 wildtype and knockout mouse over a 12:12 light-dark cycle using quantitative polymerase chain reaction. The red bars in the graph show PK2 mRNA expression in the VPAC2 wildtype (+/+) mouse which exhibits a significant diurnal oscillation with calculated peak expression ZT5:10 when fitted to a combined cosine and sine function (p-value<0.0001). The blue bars in the graph show PK2 mRNA expression in the VPAC2 knockout (−/−) mouse which is blunted and does not exhibit diurnal oscillation when fitted to a combined cosine and sine function (p-value = 0.54). Asterisks indicate statistically significant differences between the two genotypes at the indicated timepoints, as determined by two-way ANOVA followed by multiple unpaired t-tests with Bonferroni correction (p-value<0.05). Data are presented as mean ± standard error of mean (SEM). Each ZT includes data from 7 to 11 mice with a total of 52 VPAC2 +/+ (26 males/26 females) and 58 VPAC2 −/− (25 males, 33 females). PK2, prokineticin 2; SCN, suprachiasmatic nucleus; ZT, zeitgeber time.

PK2 expression exhibits diurnal oscillation with mRNA levels peaking midday, and the expression is highly dependent on functional CLOCK and BMAL1 (Cheng et al., 2002). In addition, intact VIP-VPAC2 signaling is crucial for synchronizing and maintaining SCN rhythmicity. Since almost all PK2 neurons in addition to AVP were expressing the VPAC2 receptor, we investigated by qPCR whether the diurnal expression of PK2 was intact in VPAC2 deficient mice. In VPAC2 wildtype (+/+) mice, PK2 mRNA demonstrated a highly significant diurnal expression with calculated peak ZT 5:10 (p-value<0.0001) (Figure 7). In contrast, the diurnal expression of PK2 mRNA was completely absent in VPAC2 deficient (−/−) mice (p-value = 0.54) (Figure 7). The lack of diurnal rhythmicity in PK2 mRNA expression in VPAC2−/− was primarily due to a blunted daytime expression as the significant difference in PK2 mRNA expression between the two genotypes was observed ZT4 and ZT8 (p < 0.05) (Figure 7).

### A minor population of PK2 neurons is light-responsive and directly innervated from the retina

**FIGURE 8:**
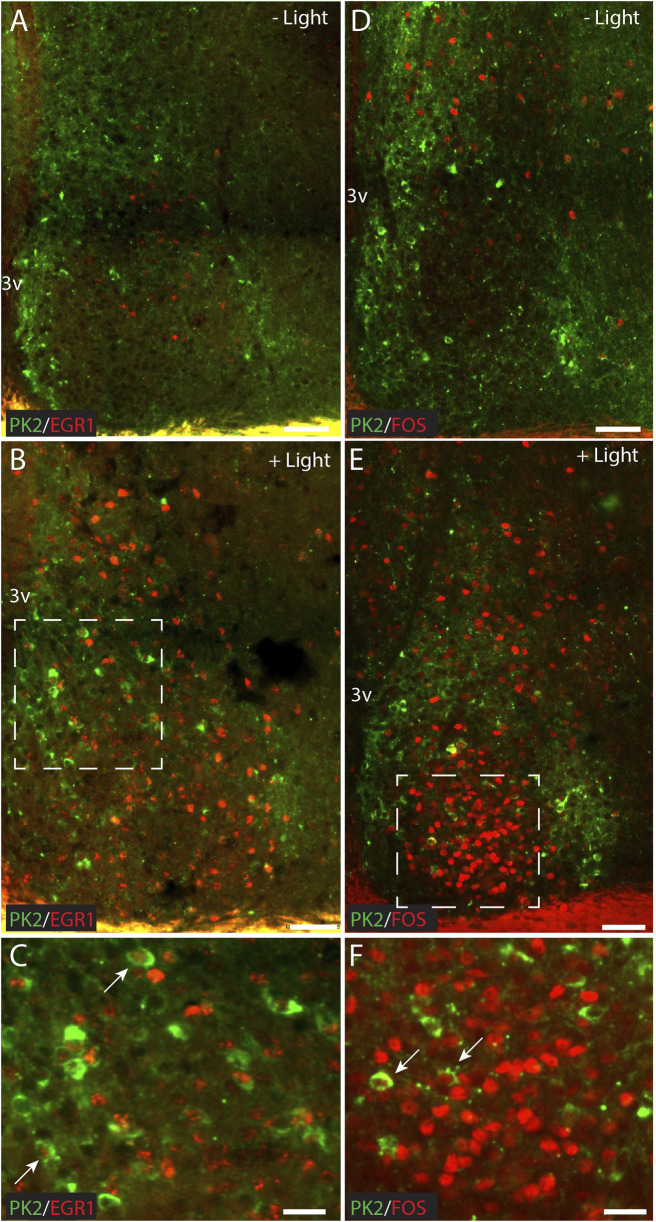
Double immunostaining for PK2 together with either FOS or EGR1 at ZT18 in the mouse SCN, with and without 30 min of light stimulation (300 lux) initiated at ZT16. **(A–C)** Show results of double immunostaining of PK2 (green) and EGR1 (red) and **(D–F)** show the results of double immunostaining of PK2 (green) and FOS (red) in the mid SCN. **(A)** Compared to **(B)** and **(D)** compared to **(E)** demonstrate that PK2 immunoreactivity is present in the SCN at ZT18, primarily in the shell and regardless of light stimulation, whereas EGR1 and FOS immunoreactivity first appear in the SCN, and primarily in the core, following light stimulation. **(C)** Represents the white frame in **(B)** whereas **(F)** represents the white frame in **(E)**. Note, that although both EGR1 **(B)** and FOS **(E)** are clearly induced in the SCN following light stimulation at night, they only seem to occur in a small subset of PK2 neurons as indicated by the white arrows in **(C,F)**. PK2, prokineticin 2; SCN, suprachiasmatic nucleus; ZT, zeitgeber time; 3V, third ventricle; vs., versus; OC, optic chiasm. Scale bars: A–B, 50 μm; C, 25 μm; D–E, 50 μm; F, 25 μm.

Light stimulation at night has been shown to induce the expression of PK2 in the SCN (Cheng et al., 2002; Cheng et al., 2005). To identify light-responsive PK2 neurons, we performed double-IHC with PK2 in combination with two immediate early gene (IEG) markers, FOS and EGR1, which are extensively expressed throughout the SCN following light-stimulation at night (Riedel et al., 2023). Two hours after a 30-min light-pulse stimulation, both FOS and EGR1 were detected throughout the rostro-caudal extent of the SCN, in both the core and shell, consistent with previous findings (Riedel et al., 2023). However, the highest density of FOS-positive neurons was in the central core, corresponding to the retinorecipient part of the SCN, whereas EGR1-positive neurons were more evenly distributed between the core and shell compartment (Figures 8B, E). Only a few neurons in which EGR1 or FOS were induced by light stimulation co-expressed PK2, with FOS-positive neurons localized in the core and EGR1-positive neurons localized in the shell (Figures 8C, F).

**FIGURE 9:**
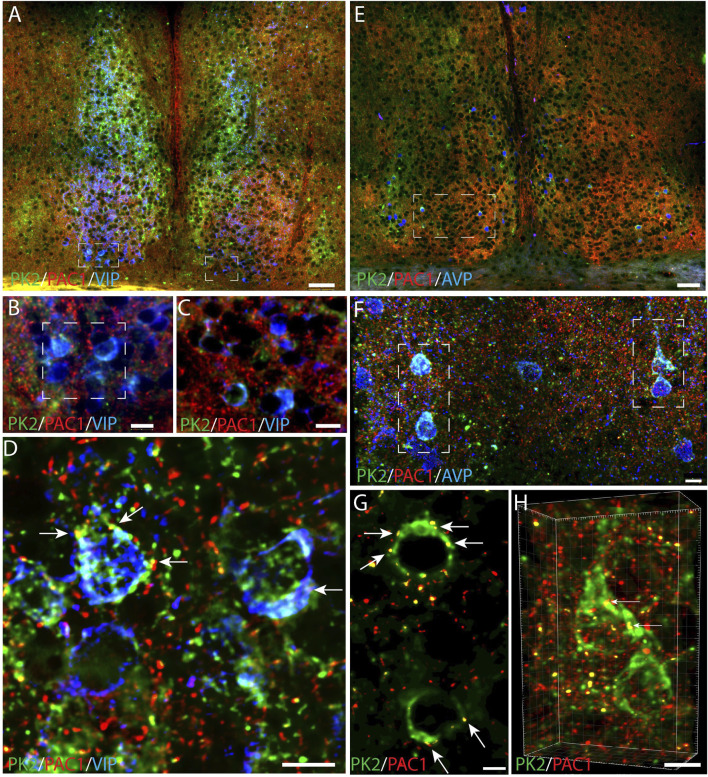
PK2, PAC1 and VIP or AVP immunoreactivity in the mouse SCN at ZT8. **(A–D)** Show results of triple immunostaining of PK2 (green), PAC1 (red) and VIP (blue). **(B,C)** represent the white frame in **(A,D)** represents the white frame in **(B)**. Together these images demonstrate that the few ventral SCN neurons in which PK2 is co-expressed with VIP also express PAC1 as indicated by the yellow dots and white arrows in **(D)**. **(E–F)** Represent triple immunostaining of PK2 (green), PAC1 (red) and AVP (blue) in the mid SCN. **(F)** Represents the white frame in **(E)** and **(G)** and **(H)** represent the white frame in **(F)** in where co-localization between PK2 and PAC1 was calculated using the IMARIS co-localization module. Co-localization is indicated by the yellow dots and highlighted by the white arrows. Image **(H)** is 3D-configurated. Note, how all PK2 neurons are a subpopulation of AVP neurons and that the PAC1 receptor is in the membrane of these neurons. PK2, prokineticin 2; PAC1, PACAP type 1 receptor, VIP, vasoactive intestinal polypeptide; AVP, vasopressin; SCN, suprachiasmatic nucleus; ZT, zeitgeber time; 3V, third ventricle; OC, optic chiasm. Scale bars: A+E, 50 μm; B, C, 10 μm; F, 8 μm; D, 7 μm; G, 4 μm; H, 5 μm.

To evaluate whether PK2 neurons receive direct retinal innervation, we additionally investigated whether PK2 neurons express the PAC1 receptor—the target for PACAP, one of the two neurotransmitters of the RHT (Hannibal, 2002). Throughout the SCN, PK2 neurons were sporadically observed to express the PAC1 receptor in the soma membrane and in proximal dendrites (Figures 9A–H). Some of these neurons constituted a minor subpopulation of VIP neurons (Figures 9A–D) and a larger subpopulation of AVP neurons (Figures 9E–H).

## Discussion

This study is the first to demonstrate, at protein level using IHC, that PK2-expressing neurons form diverse subpopulations within the SCN—primarily with AVP- and NMS-expressing neurons in the shell, and to a lesser extent, with NMS- and VIP-expressing neurons in the core—providing both qualitative and quantitative insight into the neuropeptidergic heterogeneity and topology of the SCN. Furthermore, we find the majority of PK2 neurons in the shell to co-express the VPAC2 receptor and receive dense innervation from VIP neurons located in the ventral core. In line with this, we also find that the diurnal expression of PK2 is regulated via the VPAC2 receptor, as its rhythmic mRNA expression is abolished in VPAC2 receptor-deficient mice.

RNA sequencing studies have consistently been clustering PK2, AVP and NMS within neurons of the SCN, whereas clustering of PK2 and VIP have been less definite (Morris et al., 2021; Wen et al., 2020; Chen et al., 2017). In the RNA sequencing study by Morris et al., PK2 and AVP co-expression was quantified, reporting 51% of PK2 neurons to co-express AVP (Morris et al., 2021). To date, two studies prior to ours have examined PK2 and AVP co-expression in the mouse SCN using IHC (Zhang et al., 2009; Onodera et al., 2023). However, in these previous studies, PK2 was visualized indirectly using EGFP expression in transgenic reporter models, in which 20%–60% of PK2 neurons were reported also to exhibit AVP immunoreactivity (Zhang et al., 2009). Our quantitative analysis of SCN Z-stacks double-stained for PK2 and AVP revealed that approximately 50% of PK2 neurons in the shell region of both the rostral and mid mouse SCN co-express AVP. Despite minor variability in reported percentages—potentially attributable to differences in quantification approaches—there is now consistent evidence that a substantial subset of PK2 neurons co-express AVP, and *vice versa*. Despite PK2 is typically associated with a classical shell marker, we identified a small population of PK2 neurons in the core compartment of the SCN, constituting a subpopulation of VIP neurons. When co-expression was quantified in the rostral and mid SCN, we found that approximately 20% of PK2 neurons co-expressed VIP. This contrasts with previous reports, which have consistently found less than 5% co-expression, regardless of whether RNA sequencing or IHC using EGFP as a proxy for PK2 was employed (Morris et al., 2021; Zhang et al., 2009; Onodera et al., 2023). The discrepancy in the reporting of PK2 and co-expression with other neuropeptides is likely due to differences in study design, particularly regarding the time of day at which co-expression was assessed, as the expression of most neuropeptides varies across the 24-h cycle. Since PK2 expression peaks during the mid-to-late subjective day—when both VIP and, especially, AVP levels are also high—we selected this time point to evaluate co-expression, which may strengthen the present study. Additionally, the distinction between addressing mRNA versus protein expression, along with the sensitivity of the detection systems employed, and whether the target is the PK2 protein itself or an indirect reporter protein such as EGFP may further contribute to these variations in results. It has previously been reported that NMS neurons represent ∼40% of all SCN neurons and encompass most neurons expressing AVP and VIP (Lee et al., 2015). In our study, more than 40% of NMS neurons co-expressed PK2 throughout the rostro-caudal extent of the SCN, and a comparable co-expression rate was observed for PK2 neurons co-expressing NMS. Although PK2 and NMS have consistently been clustered together in RNA sequencing studies, the extent of co-expression between these two neuropeptides has, to our knowledge, not been previously investigated (Wen et al., 2020; Chen et al., 2017). Triple-IHC combining PK2 and NMS with either VIP or AVP was not performed in this study. However, given the major co-expression between PK2 and NMS that we observed, it would be likely that the PK2 shell neuron population not only constitutes a subpopulation of AVP neurons, but a subpopulation of AVP/NMS neurons and the PK2 core neuron subpopulation not only constitutes a subpopulation of VIP neurons, but a subpopulation of VIP/NMS neurons.

PK2 and AVP are both considered output molecules from the SCN involved in transmitting circadian rhythm of behavior e.g., locomotor activity or sleep-wake rhythm and neuroendocrine functions (Mieda et al., 2015; Cheng et al., 2002; Hu et al., 2007; Kalsbeek et al., 2010; Li et al., 2018). The locomotor phenotype under different light-dark conditions, following the silencing of either a SCN neuropeptide or receptor, is a widely employed approach to elucidate the role of a neuropeptide-receptor signaling axis in the regulation of circadian rhythms. Mice lacking the vasopressin 1a receptor (V1a) and AVP-Bmal1^−/−^ mice demonstrate lengthening of the free-running period of locomotor rhythm (Mieda et al., 2015; Li et al., 2009a). A similar lengthening of the free-running period is observed in PK2^−/−^ mice (Li et al., 2006). In the study by Mieda et al., the targeted deletion of Bmal1 in AVP neurons not only eliminated AVP expression, but also PK2 expression in the shell of the SCN (Mieda et al., 2015). This finding indicates that the expression of both AVP and PK2 is clock-driven, consistent with the presence of E-box elements in their promoters (Mieda et al., 2015; Cheng et al., 2002). Moreover, the impact on PK2 expression aligns with our previously published IHC findings, demonstrating that the majority of PK2 neurons are clock-controlled, while the present study shows that these PK2 neurons constitute a subset of AVP neurons (Stangerup et al., 2025). Thus, if AVP SCN neurons are genetically manipulated (e.g., selective clock disruption, such as knockout of Bmal1), a large fraction of PK2 neurons is likely to be affected as well. Interestingly, Li et al. demonstrated an attenuation in PK2 expression in V1a^−/−^ mice, despite both the expression of AVP and clock genes remained unaffected and as in wildtype littermates (Li et al., 2009a). Conversely, the expression of AVP was unaffected in PK2^−/−^ mice (Li et al., 2006). Since the common denominator in these three knockout studies is reduced PK2 expression, the lengthening of the free-running period observed in the AVP-Bmal1^−/−^ and V1a^−/−^ mice may be the result of impaired PK2 signaling rather than impaired AVP signaling.

PK2 has been proposed to suppress daytime locomotor activity in nocturnal animals, based on its high expression during the subjective day and supported by studies showing reduced nocturnal activity following intracerebroventricular injection of PK2 (Cheng et al., 2002). However, this is not fully supported by knockout studies, as mice lacking either PK2 or its receptor, contrary to expectations, exhibit overall lower locomotor activity than their wildtype littermates (Prosser et al., 2007; Li et al., 2006). Moreover, a locomotor phenotype marked by late-night onset rather than early-night onset has been described (Prosser et al., 2007; Li et al., 2006). Furthermore, other physiological and behavioral processes such as body temperature, plasma cortisol and blood glucose as well as sleep-wakefulness exhibit attenuated circadian rhythmicity in the absence of intact PK2-PK2R signaling (Li et al., 2006; Hu et al., 2007; Li et al., 2009b). This places PK2 as a key regulator of circadian rhythmicity in the SCN, rather than merely a signaling molecule influencing locomotor activity. Hence, altered locomotor activity may serve as a general indicator of disrupted circadian rhythmicity, aligning with a recent study placing PK2 as a key modulator of SCN period and rhythmicity, shaping network dynamics to ensure stable circadian coordination (Morris et al., 2021).

Nocturnal animals respond to darkness by increasing locomotor activity (positive masking), whereas direct and immediate inhibition of locomotor activity is observed if nocturnal animals are exposed to light at night (negative masking); a process which seems to be independent of the molecular circadian clock (Hannibal, 2021). In the absence of a molecular clock, light can sustain a diurnal expression of PK2. This was demonstrated in a study by Cheng et al., as a low amplitude rhythmic PK2 mRNA expression was found in *Cry1−/−/Cry2−/−* when kept in 12:12 LD but absent when kept in 24-h darkness (DD) (Cheng et al., 2005). The double *Cry* knockout mouse displays masking-driven wheel-running activity, and PK2 has been suggested to play a role in mediating this behavior (Cheng et al., 2005; Vitaterna et al., 1999). Injection of PK2 into the lateral ventricle of rats at night rapidly suppresses locomotor activity, potentially further supporting this notion (Cheng et al., 2002).

Mice deficient of the VPAC2 receptor show no circadian clock gene expression in the SCN and exhibit wheel-running activity strictly driven by masking in LD, but become arrhythmic in wheel running activity during DD and when kept in constant light (Harmar et al., 2002; Hannibal et al., 2011; Hughes et al., 2004). In the present study, VPAC2 −/− mice exhibited complete loss of diurnal rhythmicity in PK2 mRNA expression, accompanied by significantly reduced daytime PK2 levels. Thus, our findings in VPAC2 −/− mice suggest that mechanisms other than PK2 signaling contribute to the suppression of daytime locomotor activity, underlying the negative masking effect of light observed in these animals, as VPAC2 deficiency does not result in increased wheel running activity (Hannibal et al., 2011).

In line with the abolished rhythmic PK2 expression we observed in the absence of VPAC2 receptor, a similar loss of PK2 expression has been reported in VIP-deficient mice (Bedont et al., 2018). This highlights the critical role of intact VIP-VPAC2 signaling in the regulation of PK2 expression in clock neurons located in the shell region of the SCN.

Although PK2 expression is under clock control, studies have shown that light exposure alone can sustain a low-amplitude PK2 rhythm in double cry-deficient mice in LD conditions (Cheng et al., 2005). Furthermore, light stimulation via melanopsin/PACAP-containing retinal ganglion cells (RGCs) is a key contributor to the reported nighttime light-pulse-induced expression of PK2 (Cheng et al., 2002; Cheng et al., 2005). In the present study, several observations support a role of light in regulating PK2 expression. PAC1, the PACAP-specific receptor, was localized to the soma-dendritic membrane of PK2 neurons, constituting a subpopulation of AVP-expressing neurons in the shell, and VIP-expressing neurons in the core. PACAP is one of two neurotransmitters of the RHT, suggesting direct retinal innervation of these SCN PK2 neuron subpopulations (Hannibal et al., 2017). In addition, few PK2 neurons exhibited induction of FOS or EGR1 following nighttime light stimulation. Although previous studies have shown that nocturnal light exposure induces PK2 mRNA expression in the SCN, the present study was not able to visualize an increase above basal PK2 protein levels using IHC, at least not 2 h after light-pulse stimulation. This may reflect the limited sensitivity of IHC to detect modest or transient changes in protein abundance. Thus, based on the present data, we are unable to either confirm or refuse that light induces PK2 protein expression at night.

## Conclusion

Together, our study provides new insights into the complexity of PK2 neurons in the SCN, revealing their organization into multiple subpopulations within distinct functional neuronal networks. Moreover, our findings highlight that interaction with VIP-VPAC2 signaling is fundamental for the rhythmic expression—and thereby the regulation and function—of PK2 as a putative output molecule involved in the control of circadian behavior.

## Data Availability

The raw data supporting the conclusions of this article will be made available by the authors, without undue reservation.
