# Supplementary material for: Prokineticin 2 neurons form diverse subpopulations in the suprachiasmatic nucleus and rely on VPAC2-signaling for diurnal rhythmicity
Source: Front Physiol. 2025 Jul 15;16:1619673. doi: 10.3389/fphys.2025.1619673 (PMC12303973; doi:10.3389/fphys.2025.1619673)
Supplement: Supplementary file 1 [file Supplementaryfile1.docx]

**Supplementary Table 1.** PK2 and AVP co-expression

| Animal 1 |  | Rostral SCN | Mid SCN | Caudal SCN |
| --- | --- | --- | --- | --- |
|  | PK2 (n) | 93 | 142 | 107 |
|  | AVP (n) | 128 | 183 | 184 |
|  | Co-expression (n) | 43 | 45 | 89 |
|  | AVP neurons co-expressing PK2 (%) | 34 | 25 | 43 |
|  | PK2 neurons co-expressing AVP (%) | 46 | 32 | 75 |
|  |  |  |  |  |
| Animal 2 |  |  |  |  |
|  | PK2 (n) | 67 | 94 | 142 |
|  | AVP (n) | 117 | 119 | 209 |
|  | Co-expression (n) | 43 | 62 | 89 |
|  | AVP neurons co-expressing PK2 (%) | 37 | 52 | 43 |
|  | PK2 neurons co-expressing AVP | 64 | 66 | 63 |
|  |  |  |  |  |
| Total |  |  |  |  |
|  | PK2 (n) | **160** | **236** | **249** |
|  | AVP (n) | **245** | **302** | **393** |
|  | Co-expression (n) | **86** | **107** | **169** |
|  | AVP neurons co-expressing PK2 (%) | **35** | **35** | **43** |
|  | PK2 neurons co-expressing AVP (%) | **54** | **45** | **68** |

**Supplementary Table 2.** PK2 and VIP co-expression

| Animal 1 |  | Rostral SCN | Mid SCN |
| --- | --- | --- | --- |
|  | PK2 (n) | 90 | 122 |
|  | VIP (n) | 112 | 80 |
|  | Co-expression (n) | 26 | 32 |
|  | VIP neurons co-expressing PK2 (%) | 23 | 40 |
|  | PK2 neurons co-expressing VIP (%) | 29 | 26 |
|  |  |  |  |
| Animal 2 |  |  |  |
|  | PK2 (n) | 84 | 90 |
|  | VIP (n) | 61 | 78 |
|  | Co-expression (n) | 10 | 17 |
|  | VIP neurons co-expressing PK2 (%) | 16 | 22 |
|  | PK2 neurons co-expressing VIP (%) | 12 | 19 |
|  |  |  |  |
| Total |  |  |  |
|  | PK2 (n) | **174** | **213** |
|  | VIP (n) | **173** | **158** |
|  | Co-expression (n) | **36** | **49** |
|  | VIP neurons co-expressing PK2 (%) | **21** | **31** |
|  | PK2 neurons co-expressing VIP (%) | **21** | **23** |

**Supplementary Table 3.** PK2 and NMS co-expression

| Animal 1 |  | Rostral SCN | Mid SCN | Caudal SCN |
| --- | --- | --- | --- | --- |
|  | PK2 (n) | 94 | 113 | 90 |
|  | NMS (n) | 115 | 117 | 61 |
|  | Co-expression (n) | 53 | 43 | 30 |
|  | NMS neurons co-expressing PK2 (%) | 46 | 37 | 49 |
|  | PK2 neurons co-expressing NMS (%) | 56 | 38 | 33 |
|  |  |  |  |  |
| Animal 2 |  |  |  |  |
|  | PK2 (n) | 109 | 161 | 92 |
|  | NMS (n) | 113 | 145 | 82 |
|  | Co-expression (n) | 43 | 76 | 41 |
|  | NMS neurons co-expressing PK2 (%) | 38 | 52 | 50 |
|  | PK2 neurons co-expressing NMS (%) | 39 | 47 | 45 |
|  |  |  |  |  |
| Total |  |  |  |  |
|  | PK2 (n) | **203** | **274** | **182** |
|  | NMS (n) | **228** | **262** | **143** |
|  | Co-expression (n) | **96** | **119** | **71** |
|  | NMS neurons co-expressing PK2 (%) | **42** | **45** | **50** |
|  | PK2 neurons co-expressing NMS (%) | **47** | **43** | **39** |
